# Robot Assisted Repair of Acquired Abdominal Intercostal Hernias (AIH)

**DOI:** 10.51894/001c.5964

**Published:** 2017-08-24

**Authors:** Daniel Smith, Mohan Kulkarni, Shawn Obi

**Affiliations:** 1 Henry Ford Allegiance General Surgery Resident, PGY 5, Jackson, MI; 2 Henry Ford Allegiance Thoracic Surgeon, General Surgery Program Director, Jackson, MI; 3 Henry Ford Allegiance General Surgeon, General Surgeon Teaching Facility, Jackson, M

**Keywords:** case series, laparoscopic repair, robot, intercostal hernia

## Abstract

Abdominal intercostal hernia (AIH) is a rare clinical entity in which intra-abdominal visceral contents protrude through a defect between adjacent ribs. Most AIH are repaired via (an open or a laparoscopic) transabdominal approach or a thoracotomy. In this paper, the authors present two cases of AIH. Both cases of AIH developed in male patients after severe coughing episodes and demonstrated on computed tomography (CT) to include multiple abdominal viscera. In both cases, a robot-assisted laparoscopic hernia repair was performed utilizing Sepramesh and V-Lock suturing. To our knowledge, these are the first case reports of a robotic approach to repair of AIH. Both cases demonstrate the safety of this approach and expand on novel robotic approaches to ventral hernia repairs. Studies of longer term outcomes from this surgical approach are limited in the literature due to small number of cases and even fewer associated case reports.

## INTRODUCTION

Abdominal intercostal hernias (AIH) are a rare entity with varying numbers (i.e., between 10 to 29) cases reported in the surgical literature.^1–7^ Intercostal hernias are appropriately categorized into acquired versus spontaneous with the presence (i.e., a trans-diaphragmatic intercostal hernia) or absence of diaphragmatic involvement ^1^ This case series will report on two AIH cases that did not involve the patients’ diaphragms.

Patients with AIH typically present with a symptomatic chest wall bulge in conjunction with a traumatic inciting event such as rib fracture, penetrating injury or previous surgery.^1^ Occasionally, (as in the cases reported in this paper) AIH patients present after a severe coughing episode after which they have usually been symptomatic for weeks to months.^8^ Diagnosis is usually delayed due to a low index of provider suspicion and failure to consider this entity in the differential diagnosis.^1,2^ In most cases, the AIH is formally diagnosed on a computed tomography (CT) scan of the chest and abdomen. Other diagnostic modalities include a thorough physical exam, laparoscopy, and ultrasound.

These types of hernias usually occur antero-laterally due to native weakness at the costochondral junction, a point occurring between the ribs and the costal cartilage in the front of the rib cage.^9^ Contents can include anything from within the peritoneal cavity with colon, liver, omentum, fat, and stomach all reported.^1,2^ The hernia contents are rarely incarcerated and even strangulated, in one documented case causing liver necrosis.^2^

A variety of repairs have been described in the surgical literature including open trans-abdominal, thoracotomy, and laparoscopic repair.^1^ Usually these are repaired in a tension free fashion and covered with mesh.^1^ Although some experts have advocated for re-approximation of the involved ribs, this is rarely performed due to defect size and concerns of iatrogenic rib fractures and external deformity.^1–3,6,9,10^ The recurrence of AIH is high and can be managed surgically or followed conservatively depending on the clinical context.^1,9^ The authors had found no reports of robotic repairs of an AIH.

### Presentation of Cases

Patient #1 was a Caucasian male in his mid-60’s with a history of chronic obstructive pulmonary disease (COPD), hypertension, and obesity (body mass index (BMI) of 30.43). Four months prior to evaluation, he experienced a severe coughing episode secondary to his COPD that resulted in severe right-sided chest wall pain, subsequent hematoma and contusion. Two months prior to the authors’ evaluation, the patient noticed a right antero-lateral chest wall bulge that gradually increased in size. This became increasingly symptomatic, causing him pain, nausea, and occasional emesis.

Patient #2 was a Caucasian male in his early-mid-70’s with a history of benign prostatic hypertrophy (BPH), gastro-esophageal reflux disease (GERD), obstructive sleep apnea (OSA), and obesity (BMI of 35.58). Eight months prior to evaluation, he suffered from a severe coughing episode related to an earlier hospital admission for pneumonia. The patient experienced a tearing sensation in his right anterior chest wall and gradually developed a bulge over the next few months. This bulge became increasingly symptomatic with pain during movement and insomnia due to his inability to sleep on his side.

**Figure 1:**
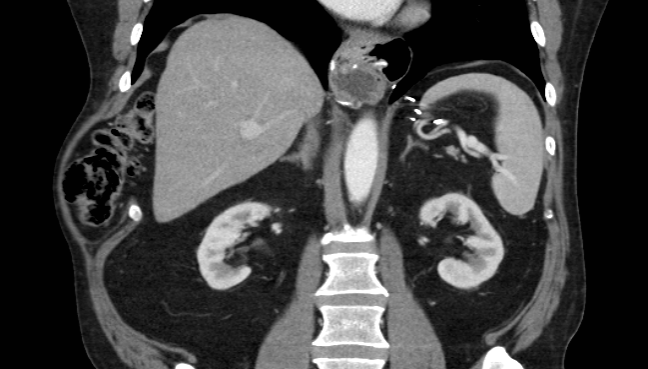
CT Scan of Patient #1 Highlighting Defect Between Anterior Ribs 9-10 and Cecum, Ascending Colon, and Omental Fat

Patient #1 was initially diagnosed with a subcutaneous lipoma. Given the persistent nature, increasing size, and worsening symptoms from the bulge, a CT scan was performed to reveal a defect between anterior ribs 9-10 including cecum, ascending colon, and omental fat (Figure 1).

**Figure 2:**
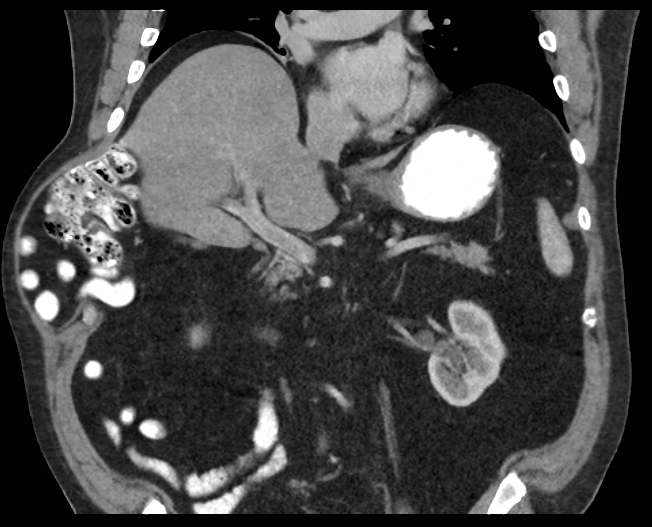
CT Scan of Patient #2 Showing a Similar Defect to Patient #1 in the Right Anterolateral Chest Wall Including Portions of Small Bowel and Portion of Right Colon

Patient #2 presented with a written report from an out of state chest CT stating the presence of an AIH. A repeat abdominopelvic CT that was performed for surgical planning revealed a similar defect to patient #1 in the right anterolateral chest wall. The hernia contents included portions of small bowel and a portion of the right colon (Figure 2).

Physical exams of both patients revealed obese males with protuberant abdomens. Lungs were bilaterally clear on auscultation with no evidence of increased work breathing. Cardiac auscultation revealed a regular rhythm with no murmurs. On both patients, right chest wall exams revealed a sizable bulge which was appropriately tender to palpation. There were no overlying cicatrices (scarring) to indicate previous trauma or surgery.

In a supine position, each of the patients’ bulges were fully reducible but immediately recurred with any patient movement. The intercostal defects were sizable on both patients with Patient #1 revealing a defect that measured approximately 6 x 11 cm. Patient #2 revealed a defect measuring approximately 11 x 13 cm. Both patients had otherwise normal abdominal examinations without features of incarceration or strangulation.

A robotic intercostal hernia repair was offered to both patients with an explanation of the possibility to convert to an open abdominal or thoracotomy approach as needed. Case #1 was performed by a thoracic surgeon and Case #2 performed by a general surgeon. Written surgical consents were obtained per protocol.

**Figure 3:**
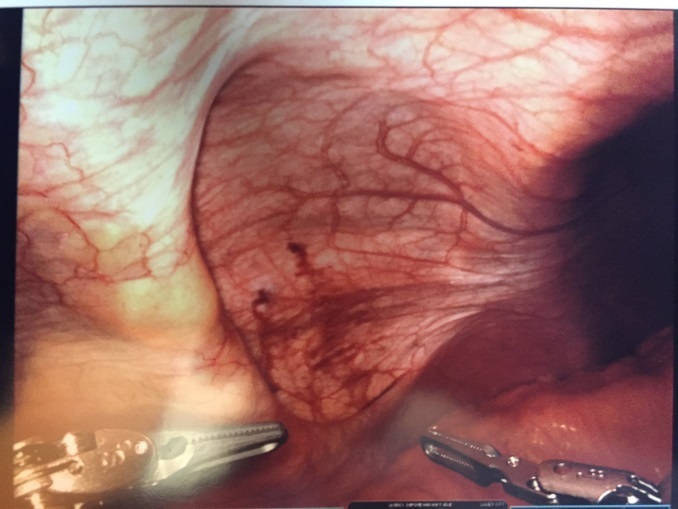
Patient #1 Hernia Contents Reduced Without Evidence of Ischemia or Perforation

Patient #1: The patient was placed in supine position, airplaned to the left at 30 degrees, and placed in a reverse Trendelenburg position (body is flat while the head is elevated 15-30 degrees higher than the feet). A peri-umbilical Veress (situated or occurring adjacent to the navel) entry was made into the peritoneal cavity and the abdomen insufflated to 15 mmHg. A peri-umbilical 8 mm. robotic trochar was placed and the abdomen inspected for any iatrogenic injuries and none were noted. Two additional 8 mm working ports were also placed, one in the right lower quadrant, and one in the epigastrium. A da Vinci surgical system Xi (Intuitive Surgical, Inc. Sunnyvale, CA) robot was then docked and the area inspected. The hernia contents were easily reduced and revealed no evidence of ischemia or perforation (Figure 3).

**Figure 4:**
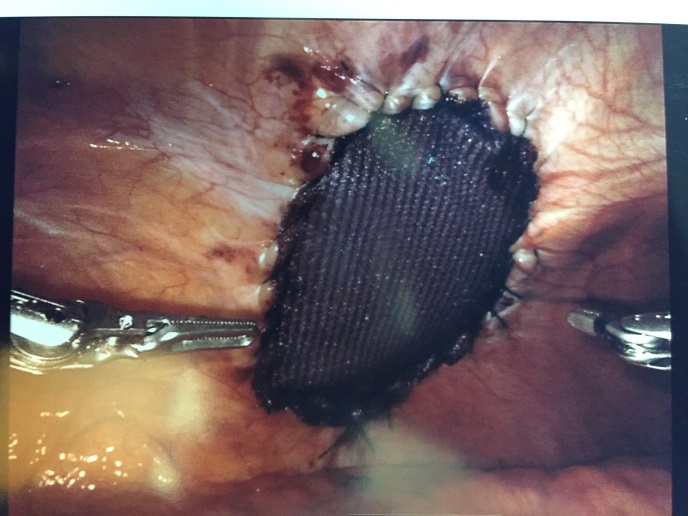
Patient #1: Mesh Affixed Circumferentially Utilizing 2-0 V-lock Suture with 2 cm. Overlap of Normal Tissue.

The intercostal defect was measured and a 7.6 x 13 cm. oval shaped Sepramesh (Bard Davol Inc. Providence RI) was brought onto the surgical field. Primary closure of the defect was not performed. The mesh was anchored in place at the antero-medial and posterolateral ends utilizing 2-0 Vicryl (Ethicon Inc, Somerville, NJ). The mesh was then affixed circumferentially utilizing a 2-0 V-lock (Medtronic, Fridley, MN) suture in a running fashion with a 2cm. overlap of normal tissue. The ribs were not incorporated in the suture (Figure 4).

The abdomen was then desufflated, the trocars removed, and the skin closed. The patient was admitted for observation, given a clear liquid diet on day of surgery, and pain controlled utilizing intravenous Tylenol and Hydromorphone. His foley catheter was discontinued upon arrival to the floor and he ambulated that day. The patient was discharged on the second post-operative day without any post-operative complications. He was doing well at his two-week post-operative office visit without pain or evidence of recurrent hernia. He did present six months postoperatively with complaints of swelling in his lateral abdominal wall. CT scan of the abdomen and pelvis revealed no evidence of AIH recurrence.

Patient #2: An open Hasson technique in the left upper quadrant was employed to gain intra-abdominal access. Three additional 8 mm working ports were placed, one in the left mid-abdomen, and one in each lower quadrant respectively. The patient was placed in reverse Trendelenburg and rotated to the left. The hernia contents were easily reduced into the peritoneal cavity. Primary closure was not performed. A 15 x 20 cm. Sepramesh was used to cover the defect and circumferentially sutured in place utilizing 2-0 V-Loc suture. The ribs or periosteum were not intentionally incorporated in the suture or mesh. Satisfactory coverage of the defect with approximately 2 cm. overlap of normal tissue was noted at the end of the case. The robot was undocked, pneumoperitoneum (abnormal presence of air or other gas) evacuated, ports removed, and the dominant 12 mm port site fascia closed utilizing O Vicryl suture.

Postoperatively he was admitted for observation with a similar pain control regimen to patient #1. His post op course was complicated by post-operative urinary retention and a hospital readmission for a post-operative ileus (malfunction in intestinal motility). These problems resolved with conservative management and supportive care. The patient was recovering well after his two-week readmission follow up appointment with no evidence of early hernia recurrence.

## DISCUSSION

AIHs are an uncommon clinical entity with fewer than 30 cases reported in the literature.^1–6^ Our two cases are interesting for several reasons. One being that there are only three case reports of a laparoscopic approach to repair this clinical entity. Our cases also appear to be the first reported repairs of an AIH utilizing robotic technology. These patients had no previous history of traumatic inciting event other than their severe coughing episodes. Despite this, both patients’ hernias were quite large containing multiple abdominal viscera.

Additionally, we utilized a V-lock suture to fixate the mesh – a facile technique using robotic technology. In both cases, we elected to not perform primary closures given the chronicity and size of the defects and low likelihood of patient benefit. In addition, primary closures may have potentially subjected these patients to unnecessary rib fractures, chronic pain from intercostal nerve entrapment, and an unsatisfactory cosmetic appearance.

To our knowledge, there are only three reports describing laparoscopic repairs of AIH defects.^3,6,10^ This is likely due to the unique and rare nature of AIH in conjunction with no standardized surgical approach. Two cases described covering the defect with an absorbable mesh with fixation using a laparoscopic tacker.^3,6^ The remaining case involved closure of the defect utilizing a percutaneous technique followed by laparoscopic placement and fixation of the mesh using a laparoscopic tacker.^10^ None of these three cases reported recurrence or complications on short term follow up. It remains unclear whether there is any benefit derived from primary closure of the defect prior to mesh placement for AIH.^2^

In 2009, a robot-assisted ventral hernia repair was first performed on a human and has been gradually gaining acceptance in the general surgery community.^11–14^ The primary purported benefit over a laparoscopic approach is the surgeon’s ability to close the fascia and use suture to fixate the mesh instead of laparoscopic tacks or transfascial stay sutures.^13^ Since there are no apparent case reports of a robotic approach to AIH repair, the overall efficacy and safety of the procedure remain undetermined. Advantages of the robotic approach include increased visualization, improved instrument dexterity, and decreased operator fatigue. Associated disadvantages include a significant capital investment, increased procedure costs, and need for specially trained support staff.

There have been several case reports of AIH from specific inciting events including trauma, rib fracture, and previous surgery.^1–3,9,10^ Additionally, there have been case reports describing AIH after an episode of severe coughing.^6,7^ This was presumably the cause of these two patients’ hernias. The sizes of both hernias were surprising large given the relatively benign inciting event. Before surgery, both these patients had suffered significantly from their hernias (e.g., primarily pain and insomnia resulting from side sleeping habits). This factor highlights the importance of a durable repair to decrease subsequent patient morbidity.

An additional advantage demonstrated in our robot-assisted cases is that the mesh could be affixed utilizing a running barbed suture, a technique which is more difficult with standard laparoscopy.^15,16^ Although technically possible with standard laparoscopy, intra-corporeal suturing is easier to master and more efficient due to the robotic wristed instruments.^17^ To our knowledge, no longer-term or prospective studies have been conducted on robotic ventral hernia repairs, making it unclear whether there is a benefit derived from using continuous sutures in place of laparoscopic tacks. It is our opinion that continuous sutures offer a more consistent approximation of the mesh to the abdominal wall, making it less likely for intra-abdominal contents to invade the pre-mesh space leading to a possible recurrence.

Although primary closures of hernia defects are routinely performed in robotic ventral hernia repairs, there are substantial differences between the anterior abdominal wall and the lateral chest wall. Our patients both suffered from their AIH for approximately six and eight months respectively, making their tissues less favorable to re-approximation. The authors concluded that both patients were at high risk for iatrogenic rib fracture or chronic pain from intercostal neuralgia if a primary closure of the defect was attempted. The external cosmetic results of primary closure were also unpredictable and were an additional reason it was not attempted. This decision coincides with several case reports describing a similar clinical outcome.^1,2,6^

It has been our practice to use Sepramesh during ventral hernia repairs due to its durability and presence of a temporary hydrogel coating on the peritoneal surface. It is our experience that the hydrogel coating decreases the likelihood of adhesions forming to the repair and causing subsequent complications. Additionally, these were both two elective cases without contamination which allowed placement of the synthetic mesh.

Ultimately, the significant contribution to the surgical community from these cases is demonstration of successful and safe AIH repair utilizing robotic technology. The approach we utilized was derived from our robot-assisted ventral hernia repair experiences. Apart from the post-operative ileus and post-operative urinary retention in Patient #2, these patients ultimately did well and benefitted significantly. The longer-term durability and safety of this surgical approach remains to be more fully elucidated in future works.

## CONCLUSIONS

AIH defects are a distinct clinical entity that can be addressed in a variety of surgical approaches. To date, a variety of open surgical techniques as well as three laparoscopic techniques have been described in the literature. We are not aware of any case reports describing a robotic approach. Robotic technology is becoming increasingly common across surgical specialties and there is an increasing need for description of novel approaches. Several points specific to our cases are worthy of further discussion and can potentially be improved upon.

The defect was not primarily closed in our cases so the durability of our repairs remains unclear. Although the benefit of running suture over tacks is speculative, our experiences indicate that this practice results in better mesh apposition. The choice of mesh was also based on our experience and comfort level. In this case series, we have described a robotic approach to repairing AIH utilizing non-absorbable mesh with a running barbed suture that appears to hold promise for future clinical applications.

### Conflict of Interest

The authors declare no conflict of interest.
